# Ocular toxoplasmosis: susceptibility in respect to the genes encoding the KIR receptors and their HLA class I ligands

**DOI:** 10.1038/srep36632

**Published:** 2016-11-09

**Authors:** Christiane Maria Ayo, Fábio Batista Frederico, Rubens Camargo Siqueira, Cinara de Cássia Brandão de Mattos, Mariana Previato, Amanda Pires Barbosa, Fernando Henrique Antunes Murata, Aparecida Perpétuo Silveira-Carvalho, Luiz Carlos de Mattos

**Affiliations:** 1 Immunogenetics Laboratory, Molecular Biology Department, Faculdade de Medicina de São José do Rio Preto. Avenida Brigadeiro Faria Lima, 5416, Vila São Pedro. CEP: 15090-000 - São José do Rio Preto, SP, Brazil; 2 FAMERP Toxoplasma Research Group, Faculdade de Medicina de São José do Rio Preto, São José do Rio Preto, SP, Brazil; 3 Ophthalmology Outpatient Clinic, Hospital de Base de São José do Rio Preto, Fundação Faculdade Regional de Medicina de São José do Rio Preto. Avenida Brigadeiro Faria Lima, 5544, Vila São José. CEP: 15090-000 - São José do Rio Preto, SP, Brazil

## Abstract

The objective of this study was to investigate the influence of the genes encoding the KIR receptors and their HLA ligands in the susceptibility of ocular toxoplasmosis. A total of 297 patients serologically-diagnosed with toxoplasmosis were selected and stratified according to the presence (n = 148) or absence (n = 149) of ocular scars/lesions due to toxoplasmosis. The group of patients with scars/lesions was further subdivided into two groups according to the type of ocular manifestation observed: primary (n = 120) or recurrent (n = 28). Genotyping was performed by PCR-SSOP. Statistical analyses were conducted using the Chi-square test, and odds ratio with a 95% confidence interval was also calculated to evaluate the risk association. The activating *KIR3DS1* gene was associated with increased susceptibility for ocular toxoplasmosis. The activating KIR together with their HLA ligands (KIR3DS1-Bw4-80Ile and KIR2DS1^+^/C2^++^ KIR3DS1^+^/Bw4-80Ile^+^) were associated with increased susceptibility for ocular toxoplasmosis and its clinical manifestations. KIR-HLA inhibitory pairs -KIR2DL3/2DL3-C1/C1 and KIR2DL3/2DL3-C1- were associated with decreased susceptibility for ocular toxoplasmosis and its clinical forms, while the KIR3DS1^−^/KIR3DL1^+^/Bw4-80Ile^+^ combination was associated as a protective factor against the development of ocular toxoplasmosis and, in particular, against recurrent manifestations. Our data demonstrate that activating and inhibitory *KIR* genes may influence the development of ocular toxoplasmosis.

Ocular Toxoplasmosis, the most common form of posterior uveitis, results from *Toxoplasma gondii* infection^1^. The prevalence varies widely between different countries however, both the frequency and the severity of the resulting ocular manifestations are higher in Brazil than in many other parts of the world^2^,^3^. Eye injuries affect the retina and the choroid with local inflammatory reactions being observed in ocular tissues infected by *T. gondii*^1^.

The damage to ocular tissues led to the proposal of phenomena that may be related to pathogenic mechanisms of ocular toxoplasmosis, including autoimmune mechanisms^1^,^4^. Now it is known that an exaggerated T-helper 1 (Th-1) response, in particular by Th-17 cells, can cause tissue damage and contribute to the severity of ocular toxoplasmosis due to the production of interleukin-17 (IL-17), a potent inducer of inflammation^5^,^6^. In addition to Th-17, other sources of IL-17, including natural killer cells (NK), can contribute to the development of inflammatory conditions^7^.

Increases in the numbers of circulating proinflammatory monocytes and NK CD56^dim^ cytotoxic cells and a decrease in immunoregulatory NK CD56^bright^ cells have been identified in children congenitally infected with *T. gondii* who have active eye lesions. Furthermore, subsets of NK cells and CD8^+^ T cells play a crucial role as biomarkers of cicatricial lesion of the eye^8^. There is also evidence that NK cells have a predominantly proinflammatory profile *in vitro* during *T. gondii* infections, due to an increased production of interferon-gamma (IFN-γ) in patients with congenital ocular toxoplasmosis^6^.

The effector function of NK cells is regulated by a set of receptors named killer immunoglobulin-like receptors (KIR) expressed on the cell surface that recognize human leukocyte antigen (HLA) class I molecules of target cells^9^,^10^. *KIR* genes are responsible for coding the KIR receptors of NK cells. These genes comprise a family of 15 genes located on chromosome 19q13.4 characterized as inhibitors (*KIR2DL1*, -*2DL2*, -*2DL3*, -*2DL5A,-2DL5B* -*3DL1*, -*3DL2*, and -*3DL3*) or activators (*KIR2DS1*, -*2DS2*, -*2DS3*, -*2DS4*, -*2DS5*, -*3DS1* and -*2DL4*,), and two pseudogenes (*KIR2DP1* and -*3DP1*). Moreover, based on the content of the genes, KIR genotypes are divided into two groups designated AA and BX (BB and AB) that differ in the number and type of *KIR* genes^11^.

*KIR* genes have been described as risk or protective factors in different types of non-toxoplasmic uveitis and inflammatory ocular diseases. These diseases include Behcet’s uveitis^12^, uveitis in patients with spondyloarthropathies^13^,^14^ and Vogt-Koyanagi-Harada syndrome^15^,^16^, all of which are triggered by autoimmune processes. *KIR* genes are also associated with many other infectious diseases^17^,^18^,^19^. Additionally, both murine and human studies have shown that major histocompatibility complex (MHC) class I (called HLA class I in human) are associated with Toxoplasma susceptibility^20^,^21^,^22^,^23^,^24^,^25^,^26^.

NK cells have great importance in the control of *T. gondii* infection^27^ however, the role of *KIR* genes that encode the immune receptors of NK cells and can trigger local inflammation in the eye has not been elucidated in ocular toxoplasmosis yet. The objective of this study was to investigate the influence of the genes encoding the KIR receptors and their HLA ligands in the resistance or susceptibility to the development of ocular toxoplasmosis.

## Results

### General characteristics of patients with and without ocular manifestations of toxoplasmosis

The characteristics of the study population with respect to age, gender, clinical diagnosis and serological profile are shown in Table 1. The median ages were significantly different between the groups: A higher mean age was observed for the group of patients without ocular toxoplasmosis compared to the group of patients with ocular toxoplasmosis (*P* < 0.0001, t = 7.00), with the subgroup of patients with primary manifestations of ocular toxoplasmosis (*P* < 0.0001; t = 5.48) and with the subgroup of patients with the recurrent form of the disease (*P* < 0.0001; t = 7.51). A higher mean age was also observed for the subgroup of patients with primary manifestations than those who had recurrent manifestations (*P* = 0.002; t = 3.12).

### Distribution of KIR genes and KIR genotypes in patients with and without ocular toxoplasmosis

The distribution of genotype frequencies of *KIR2DL2/3* and *KIR3DL1/S1* were in Hardy–Weinberg equilibrium (*P* > 0.05) in this study population. However, *KIR3DL1/S1* for the patient group that developed ocular toxoplasmosis was not in Hardy-Weinberg equilibrium (*P* = 0.03). *KIR* framework genes, *KIR2DL4, KIR3DL2, KIR3DL3* and *KIR3DP1*, as expected were present in all samples, which are important internal controls to check the quality of genotyping.

The distribution of *KIR* gene frequencies and AA and BX genotype frequencies are shown in Table 2. An increased susceptibility for developing ocular toxoplasmosis (OR = 2.15; CI = 1.31–3.50; *P* = 0.003, *Pc* = 0.04) was observed for the *KIR3DS1* activating gene. There was also a positive association between *KIR3DS1* and recurrent manifestations of disease (OR = 3.25; CI = 1.42–7.44, *P* = 0.007; *Pc* = 0.1) when this patient group was compared to patients without ocular toxoplasmosis, although it was lost after applying the Bonferroni correction. On the other hand, the *KIR2DS2* activating gene was associated with decreased susceptibility for ocular toxoplasmosis (OR = 0.55; CI = 0.31–0.97; *P* = 0.05; *Pc* = 0.80), but the significance was also lost after applying the Bonferroni correction. No significant difference was observed in the AA and BX genotype frequencies between all groups investigated in this study.

### Distribution of the HLA class I KIR-ligands in in patients with and without ocular toxoplasmosis

Human leucocyte antigen frequencies was in Hardy-Weinberg equilibrium (*P* > 0.05) in all groups studied. The frequencies of the HLA class I ligands of KIR (A3 or A11, Bw4–80Ile and −80Thr, C1 and C2, in homozygosity and heterozygosity) were analyzed and were similar between groups (Table 3).

### Distribution of KIR and their respective HLA ligands in patients with and without ocular toxoplasmosis

Data on the distribution of KIR genes with their HLA class I ligands are listed in Table 4. The KIR3DS1-Bw4-80Ile pair was associated to increased susceptibility for developing ocular toxoplasmosis. The frequency was higher for patients with ocular toxoplasmosis (OR = 2.28; CI = 1.24–4.19; *P* = 0.007; *Pc* = 0.01), patients with primary manifestations (OR = 2.08; CI = 1.09–3.95; *P* = 0.02; *Pc* = 0.04) and patients with recurrent manifestations (OR = 3.24; CI = 1.28–8.89; *P* = 0.02; *Pc* = 0.04) than patients without ocular toxoplasmosis.

The *KIR2DL3* inhibitory allele in the homozygous state and presence of its ligands whether homozygous or not (KIR2DL3/2DL3-C1/C1 and KIR2DL3/2DL3-C1) was associated with resistance to ocular toxoplasmosis when patients without ocular toxoplasmosis were compared with those with ocular toxoplasmosis (OR = 0.19; CI = 0.07–0.51; *P* = 0.0005; *Pc* = 0.001 and OR = 0.27; CI = 0.15–0.48; *P* = 0.000007; *Pc* = 0.00002 respectively), primary manifestations (OR = 0.23; CI = 0.08–0.64; *P* = 0.002; *Pc* = 0.006 and OR = 0.31; CI = 0.17–0.56; *P* = 0.0001; *Pc* = 0.0003 respectively) and recurrent manifestations (OR = 0.09; CI = 0.05–0.91; *P* = 0.01; *Pc* = 0.05 and OR = 0.12; CI = 0.29–0.59; *P* = 0.003; *Pc* = 0.009 respectively) (Table 4).

### Frequencies of the number of KIR-HLA class I activating and inhibitory ligands in patients with and without ocular toxoplasmosis

**Figure 1:**
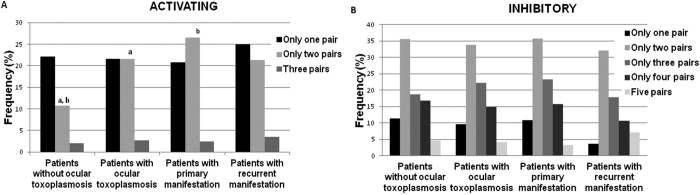
Frequencies of pairs of KIR-HLA class I activating (**A**) and inhibitory (**B**) ligands in patients with and without ocular toxoplasmosis and its manifestation as primary or recurrent. ^a^OR = 2.29; CI = 1.19–4.39; *P* = 0.01; *Pc* = 0.04 (Patients without ocular toxoplasmosis vs. Patients with ocular toxoplasmosis); ^b^OR = 2.29; CI = 1.16–4.52; *P* = 0.01; *Pc* = 0.04 (Patients without ocular toxoplasmosis vs. Patients with primary manifestation).

Figure 1 shows the influence of the number of KIR-HLA class I activating and inhibitory ligands on the development of ocular toxoplasmosis and its clinical manifestations. There were significant differences between patients with and without ocular toxoplasmosis (OR = 2.29; CI = 1.19–4.39; *P* = 0.01; *Pc* = 0.04) and patients with primary manifestations and without ocular toxoplasmosis (OR = 2.29; CI = 1.16–4.52; *P* = 0.01; *Pc* = 0.04) when only two pairs of activating ligands were present (Fig. 1A). Significant associations were not found in the analysis of pairs of inhibitory KIR-HLA class I ligands (Fig. 1B).

**Figure 2:**
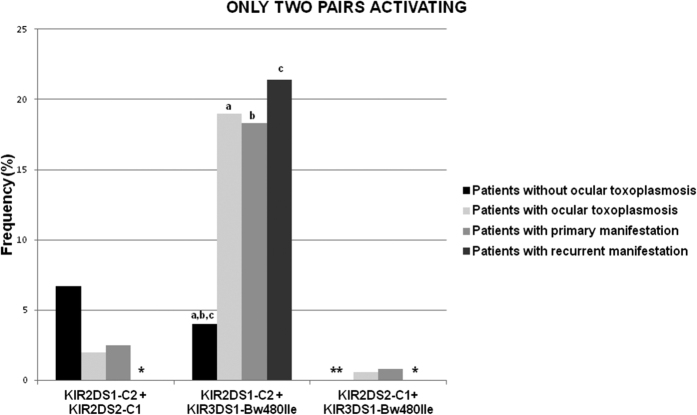
Distribution of the frequencies of the possible combinations of only two KIR activating genes in the presence of their HLA Class I ligands in patients with and without ocular toxoplasmosis and its manifestations as primary or recurrent. * and ** represents respectively that patients with recurrent manifestations and patients without ocular toxoplasmosis presented results of 0% for two activating pairs. ^a^OR = 5.56; CI = 2.22–13.88; *P* = 0.0001; *Pc* = 0.0004 (Patients without ocular toxoplasmosis vs. Patients with ocular toxoplasmosis); ^b^OR = 5.35; CI = 2.09–13.68; *P* = 0.0002; *Pc* = 0.0008 (Patients without ocular toxoplasmosis vs. Patients with primary manifestations); ^c^OR = 6.50; CI = 1.92–21.96; *P* = 0.004; *Pc* = 0.01 (Patients without ocular toxoplasmosis vs. Patients with recurrent manifestations).

Subsequently, discrimination of the two activator pairs (KIR-HLA class I) for the association mentioned above was investigated. The combination involving KIR2DS1 and KIR3DS1 in the presence of their respective ligands (KIR2DS1^+^/C2^++^ KIR3DS1^+^/Bw4–80Ile^+^) is responsible for increasing the risk of developing ocular toxoplasmosis (OR = 5:56; CI = 2.22–13.88; *P* = 0.0001; *Pc* = 0.0004). The combination is also responsible for its primary (OR = 5.35; CI = 2.09–13.68; *P* = 0.0002; *Pc* = 0.0008) and recurrent clinical forms (OR = 6.50; CI = 1.92–21.96; *P* = 0.004; *Pc* = 0.01) compared to patients without ocular toxoplasmosis (Fig. 2).

### Distribution of activating KIR plus inhibitory KIR and their respective ligands in patients with and without ocular toxoplasmosis

The correlation between the distribution of activating and inhibitory KIR and their respective HLA ligands was analyzed (Table 5). A decreased risk of developing ocular toxoplasmosis (OR = 0.52; CI = 0.32–0.84; *P* = 0.009; *Pc* = 0.02) and recurrent manifestations of the disease (OR = 0.13; CI = 0.03–0.45; *P* = 0.0003; *Pc* = 0.0009) was observed for the KIR3DS1^−^/KIR3DL1^+^/Bw4–80Ile^+^ combination (KIR2DL1 and the Bw4-80Ile ligand in the absence of KIR3DS1) when compared to patients without ocular toxoplasmosis. This correlation was also observed when patients with recurrent manifestations were compared to patients with primary manifestations (OR = 0.20; CI = 0.05–0.70; *P* = 0.006; *Pc* = 0.01).

## Discussion

Possible causes of ocular manifestations of toxoplasmosis have not been fully elucidated, but it is believed that factors related both to the parasite and the host contribute to the development of this disease^4^,^28^. Therefore, the study of the impact of genes on infections, such as toxoplasmosis, is extremely important because it provides information about the contribution of the host’s genetic factors on the development of this type of disease. We have previously shown that MICA polymorphisms are not associated with the development of ocular toxoplasmosis^29^. Using the same samples, the current study found that KIR receptors genes and their HLA ligands are associated with the development of ocular lesions resulting from *T. gondii* infection. To the best of our knowledge, this is the first study of *KIR* genes and HLA ligands in the immunopathology of ocular toxoplasmosis.

The difference in the mean ages of the patient groups of this study was carefully discussed previously^29^. Briefly, *T. gondii* infection can occur at any time of life, and although most cases of ocular toxoplasmosis occur due to infections acquired after birth, a significant number of patients can acquire the disease congenitally and the resulting scars trend to be persistent^1^. It has also been demonstrated that the risk of recurrence is higher in the year following the first infection than in future years^30^,^31^. As no distinction was made between congenital and acquired disease in the analysis of the characteristics of eye injuries, this may be one of the possible explanations for the lower mean age observed for patients who developed ocular toxoplasmosis, including those who present with recurrent signs of the disease. Furthermore, other eye diseases, those without scars/lesions due to toxoplasmosis, are prevalent in older patients^32^.

Regarding the distribution of *KIR* genes, after Bonferroni correction only the *KIR3DS1* activating gene was associated with increased risk of developing ocular toxoplasmosis with the other associations being lost. The Bonferroni correction decreases the chance of a significant difference by chance alone, making the data more robust^33^. The association observed for the *KIR3DS1* with ocular toxoplasmosis can be explained by the absence of *KIR3DL1*, because *3DS1* and *3DL1* segregate as alleles of a single locus. Thus, the presence of *KIR3DS1*, and consequently the absence of *KIR3DL1*, can create an increased potential for the activation of NK cells owing to decreases in the ratio of inhibitory/activating receptors. Previous studies have shown involvement of the *KIR3DS1*/*L1* alleles in various types of non-toxoplasmic uveitis and inflammatory eye diseases triggered by autoimmune factors. Levinson *et al*.^15^ observed that individuals with the *KIR3DS1* gene in their haplotype have an increased risk of developing Vogt-Koyanagi-Harada syndrome, while the presence of *KIR3DL1* was associated to protection against the development of the disease. A similar result was observed by Moon *et al*.^14^ for the development of uveitis related to ankylosing spondylitis: *KIR3DS1* was associated as a risk factor and *KIR3DL1* was associated as a protective factor.

In this study, *KIR3DL1/S1* for the patient group that developed the ocular toxoplasmosis were not in Hardy-Weinberg equilibrium. However, some authors claim that this equilibrium should only be investigated in the control group, because it represents the general population^34^,^35^. Thus, the high frequency of *KIR3DS1* might be changing the distribution of these alleles in individuals with ocular toxoplasmosis, resulting in a deviation from the Hardy-Weinberg equilibrium. Considering that numerous precautions were adopted to prevent bias in this study, we can safely say that the *3DS1* gene exerts a real influence on the development of ocular toxoplasmosis, even though *KIR3DL1/S1* were not in Hardy-Weinberg equilibrium.

The function of *KIR* genes in immune response is highly dependent on the HLA molecules expressed on the target cell surface. Therefore, KIR receptors influence susceptibility for or protection against certain illnesses by means of a balance in activation and inhibition signals that regulate the NK cell effector function^9^,^10^. The recognition of specific HLA ligands by inhibiting KIR is well established^36^. There is clear evidence that HLA-Bw4-80Ile is powerfully recognized by KIR3DL1, but there is still controversy as to whether its homologue, KIR3DS1, interacts with the same ligand, as only indirect evidence was found^37^,^38^. There is also a hierarchy of inhibition related to KIR2DL receptors in which KIR2DL3-C1 has lower inhibitory potential than KIR2DL2-C1 and KIR2DL1-C2^39^.

In this study, where the *KIR* genes were analyzed in the presence of their respective ligands (KIR-HLA), the KIR3DS1-Bw4-80Ile pair was associated with the development of ocular toxoplasmosis irrespective of the type of clinical manifestation (primary or recurrent); while KIR2DL3/2DL3-C1/C1 and KIR2DL3/2DL3-C1 were associated with protection against the development of ocular toxoplasmosis and its clinical manifestations. In accord with our results, other studies found an association involving the KIR3DS1-Bw4-80Ile pair in other human diseases^40^,^41^,^42^,^43^, suggested an interaction between these molecules. *KIR2DL3* and *KIR2DL2* are considered alleles, as are *KIR3DL1* and *KIR3DS1*. Although the KIR2DL2-C1 interaction is stronger than the KIR2DL3-C1 interaction, the inhibitory signal generated by the absence of KIR2DL2 (KIR2DL3/2DL3-C1/C1 and KIR2DL3/2DL3-C1) appears to be sufficient to inhibit the effector function of NK cells and protect against elevated inflammation and tissue damage. It has been shown that abnormal expressions of inhibitory receptors of NK cells, including the KIR2DL3 receptor, may be associated with the development of Behcet’s disease^44^.

It is important to highlight the results observed for the number of pairs of KIR-HLA ligands and the correlation between the distribution of activating KIR and their respective HLA ligands. A higher frequency of only two pairs of ligands was observed in patients with ocular toxoplasmosis and in patients with primary manifestations compared to patients without ocular toxoplasmosis. The combination responsible for this association was KIR2DS1^+^/C2^++^ KIR3DS1^+^/Bw4-80Ile^+^, although the *KIR2DS1* and *KIR3DS1* genes are not in linkage disequilibrium as observed among patients with ocular toxoplasmosis (Δ′ = 0.06; *P* = 0.68) and patients without ocular toxoplasmosis (Δ′ = 0.15; *P* = 0.45). The combination of one pair (inhibitory) in the absence of the other pair (activating) was analyzed. It was possible to observe that the KIR3DS1^−^/KIR3DL1^+^/Bw4-80Ile^+^ combination decreases the risk of developing ocular toxoplasmosis and its recurrent clinical forms.

These results may suggest that the activating function mediated by KIR2DS1 plus KIR3DS1 and their respective HLA ligands is, in fact, an important factor interfering in ocular toxoplasmosis, since such a combination may affect the balance of inhibiting/activating signals. NK cells are activated when there is an increase of activation signals, even if there is a combination of strong or weak inhibitory signals^45^. Yet, the absence of KIR3DS1 in the presence of its inhibitor homologue, KIR3DL1, was indicative of the inhibitory or protective role played by NK cells, which perhaps avoids subsequent immune responses that may trigger inflammation and autoimmunity. In support of our findings, Levinson *et al*.^46^ demonstrated both negative and positive associations mediated by KIR-HLA pairs in another ocular inflammatory disease, birdshot chorioretinopathy.

NK, CD4 and CD8 T cells, and type 1 cytokines, such as IFN-γ and IL-2, play a protective role in *T. gondii* infection. IL-12 stimulates NK cells to produce IFN-γ and to promote the development of Th-1 cells that produce IFN-γ, a cytokine involved in the activation of macrophages, the main phagocytes in chronic inflammation^47^,^48^. In the eye, although the immune response is usually suppressed to prevent tissue damage, there is experimental evidence that *T. gondii* infection promotes the production of factors such as IFN-γ that suppress the immune privilege of this organ^49^. This possibly leads to increased severity of lesions with marked necrosis or inflammation of the retina and the choroid^50^,^51^.

The immune response can determine the development of eye injuries resulting from *T. gondii* infection, and the mechanisms involved may be associated with both the pathogenesis and protective effects that control tissue damage. It has been shown that increases in the frequency of circulating NK cells and proinflammatory monocytes in children infected by *T. gondii*, particularly in those with active ocular lesions, are indicative of a strong and persistent proinflammatory response. Moreover, subsets of NK cells and CD8^+^ T cells act as biomarkers for cicatricial lesions of the eye^8^. It has also been demonstrated that NK cells show increased production of IFN-γ in patients with congenital ocular toxoplasmosis^6^. Furthermore, the high imunopathogenicity responsible for tissue damage and deterioration of ocular toxoplasmosis may be due to the presence of a potent inducer of inflammation, IL-17^5^,^6^, which is also produced by NK cells^7^. On the other hand, ocular toxoplasmosis may be linked to autoimmunity^1^,^4^.

The current study investigated the KIR-HLA ligand as a risk factor in ocular toxoplasmosis and these results may improve to understanding of the immunopathogenic mechanism involving NK cells in ocular manifestations related to toxoplasmosis. However, others studies should be performed such as histological analyses of the ocular tissue affected by *T. gondii* and NK cytotoxicity assays to better understanding the role of NK cells and the expression of KIR in the immunopathogenesis of ocular toxoplasmosis.

In conclusion, the results of this study show that activating and inhibitory KIR in the presence of their respective HLA ligands may have influence on the development of ocular toxoplasmosis and its clinical form in this population. In particular this is seen with the strong presence of activating signals as risk factors (*KIR3DS1*, KIR3DS1-Bw4-80Ile and KIR3DS1^+^/Bw4-80Ile^++^ KIR2DS1^+^/C2^+^) and inhibitory signals as protective factors (KIR2DL3/2DL3-C1, KIR2DL3/2DL3-C1/C1 and KIR3DS1^-^/KIR3DL1^+^/Bw4-80Ile^+^).

## Methods

The design of this study aimed to follow, as close as possible, the criteria recommended by STrengthening the REporting of Genetic Association Studies (STREGA)^52^.

### Ethics Information

This study was approved by the Research Ethics Committee of the Medicine School in São José do Rio Preto (#1980/2009) and all individuals who agreed to participate signed informed consent forms. The experiments were carried out in accordance with the approved relevant guidelines and regulations.

### Patient selection

A total of 297 unrelated patients from the Retinopathy Outpatient Service of Hospital de Base of the Medicine School in São José do Rio Preto (HB-FUNFARME) and Medical Outpatient Clinic (AME) in São José do Rio Preto participated in this study.

The study subjects have been described previously^29^. Patients were grouped according to the presence of ocular scars/lesions due to toxoplasmosis (n = 148; 79 men and 69 women; mean age: 42.3 ± 20.6 years) or to the presence of ocular diseases not related to toxoplasmosis (n = 149; 73 men and 76 women; mean age: 57.7 ± 16.9 years). The group of patients with scars/lesions due to toxoplasmosis was further subdivided into two groups according to the type of ocular manifestation observed during a follow up period of at least two years: primary manifestations (n = 120; 65 men and 55 women; mean age: 44.9 ± 20.9 years) and recurrent manifestations characterized by the presence of satellite lesions^53^ (n = 28; 14 men and 14 women; mean age: 31.8 ± 30.5 years) (Table 1).

All individuals who participated in this study were monitored and evaluated in respect to clinical symptoms, serology for *T. gondii* and epidemiological data. Besides, although patients self-reported themselves as European descent, mixed African and European descent, and African descent, due to high miscegenation of the Brazilian population they were defined as a population of mixed ethnicity^54^.

In this study, in order to avoid bias in the results, all patients were selected after clinical examination using the same criteria. Additionally, the probability of variations in the allele frequencies due to ethnic background was minimized by matching patients with ocular toxoplasmosis and patients without ocular toxoplasmosis from similar ethnic backgrounds. Furthermore, gender and residence in the same geographical areas were carefully matched during group selection.

The number of patients enrolled is sufficient to demonstrate whether there is an association between ocular toxoplasmosis and *KIR* genes with statistical power of more than 90% and it was chosen according to frequency of *KIR* genes recorded in Allele*Frequencies database (http://www.allelefrequencies.net) observed in a population located in the southeast region of Brazil and defined as a population of mixed ethnicity.

### Inclusion/exclusion criteria

The inclusion criteria of patients with ocular toxoplasmosis were positive laboratory diagnosis of toxoplasmosis, the presence of ocular scars/lesions due to toxoplasmosis and live in municipalities in the northwest region of the State of Sao Paulo (located in the southeast region of Brazil, between 20°49'13″S and 49°22′47″W). The inclusion criteria of patients without ocular toxoplasmosis were positive laboratory diagnosis of toxoplasmosis but without ocular scars/lesions due to toxoplasmosis and living in the same geographical region as the patients with ocular toxoplasmosis. All patients were clinically evaluated by two experienced physicians.

The exclusion criteria were: patients with other infectious and parasitic diseases, patients with any type of mental disability, patients with blood dyscrasia and using oral anticoagulants and related patients.

### Laboratory diagnosis

Blood samples were collected into tubes without anticoagulant to obtain serum. *A*nti*-T. gondii* antibodies were detected by immunosorbent assay (ELISA) according to the manufacturer’s instructions (ETI-TOXOK-M reverse PLUS; DiaSorin S.p.A. Italy and ETI-TOXO-G PLUS; DiaSorin S.p.A. Italy). The microplates were read using Epoch™ equipment using the Gen5™ 2.0 software (BioTek, Winooski, Vermont, USA). The samples were tested in duplicate and in cases of indeterminate results, the samples were retested in duplicate. Positive and negative controls were included in all reactions.

### Clinical diagnosis

All patients were clinically assessed using an indirect binocular ophthalmoscope (Binocular Ophthalmoscope ID10, Topcon Corporation, USA). The evaluation of visual acuity followed the logMAR Early Treatment Diabetic Retinopathy Study chart (ETDRS) criteria^55^. Intraocular pressure was measured by Goldmann applanation tonometry, and stereoscopic biomicroscopy was performed using a 78-diopter lens (Volk) and a slit lamp and all they were classified according to the ETDRS criteria^55^.

As no invasive test was performed, the ocular toxoplasmosis diagnostic criteria used were the same as in the clinical practice: injury identified by ophthalmoscopy associated with positive serology for *T. gondii*. This is therefore a presumptive diagnosis.

### KIR and HLA genotyping

Blood samples were also collected into tubes containing EDTA anticoagulant for DNA extraction. DNA of all patients was extracted using the commercial kit for silica column extraction (QIAamp^®^ DNA Blood Mini Kit, QIAGEN, the Netherlands) following the manufacturer’s instructions. All DNA samples were subjected to an evaluation of concentration and purity using the ratio of the absorbance at optical densities (OD) of 260 and 280 nm with Epoch™ equipment (BioTek, Winooski, Vermont, USA). KIR and HLA-A, -B and -C were genotyped according to manufacturer’s instructions by Polymerase chain reaction-sequence specific oligonucleotide probe (PCR-SSOP) protocols with Luminex^®^ technology (One Lambda Inc., Canoga Park, CA, USA). This technique uses PCR-amplified DNA with specific biotinylated primers. The amplified product is hybridized by complementary DNA probes conjugated to fluorescently coded microspheres, with detection using R-Phycoerythrin-conjugated Streptavidin (SAPE). The data were interpreted using a computer program (HLA Fusion, 2.0 Research, One Lambda).

KIR2DL1 and KIR2DS1 bind to HLA molecules from the C2 group, which include the HLA-C*02, *04, *05, *06, *07,*15, *17, and *18 specificities. KIR2DL2, KIR2DL3 and KIR2DS2 interact with HLA molecules from the C1 group, among them: HLA-C*01, *03, *07, *08, *12, *13, *14 and *16. KIR3DL2 binds to HLA-A*03 or -A*11 specificities and KIR3DL1 recognizes HLA-Bw4 epitopes (HLA-A*23, *24, *25, *32; HLA-B*13, *27, *44, *51, *52, *53, *57, *58). HLA-Bw4 molecules were divided into two groups based on whether isoleucine or threonine was present at position 80 (Bw4-80Ile and Bw4-80Thr). KIR3DS1 binds to Bw4-80Ile molecules. HLA-KIR ligand specificities were considered according to Carr *et al*.^37^, Thananchai *et al*.^56^ and Kulkarni *et al*.^57^.

Two types of KIR genotypes have been described based on the content of the genes: AA and BX (BB and AB) (defined according to http://www.allelefrequencies.net). Individual genotypes were determined to be AA when the genes *KIR2DL1, KIR2DL3, KIR2DL4, KIR2DS4, KIR3DL1, KIR3DL2, KIR3DL3, KIR2DP1* and *KIR3DP1* were present. The presence of one or more of the following genes: *KIR2DL5, KIR2DS1, KIR2DS2, KIR2DS3, KIR2DS5* and *KIR3DS1* characterized the BX genotype.

### Statistical analysis

KIR, HLA and KIR-HLA frequencies were obtained by direct counting. The comparisons of the frequencies of HLA ligands, KIR genes, KIR AA and BX genotypes and KIR with or without ligands between groups of patients were performed with the Chi-square test with Yates’ correction or, when necessary, Fisher’s exact test using the program Graph Pad Instat (http://www.graphpad.com/quickcalcs/contingency1.cfm). Odds ratio (OR) with a 95% confidence interval (95% CI) was also calculated to evaluate the risk association. The mean ages were compared using the t-test. Differences with *P*-values < 0.05 corrected by the Bonferroni inequality method for multiple comparisons (*Pc*) were considered statistically significant. A Hardy-Weinberg equilibrium fit was performed by calculating expected genotype frequencies and comparing that with the observed values for KIR2DL2/3, KIR3DL1/S1, and the HLA alleles using ARLEQUIN software, version 3.1. (http://cmpg.unibe.ch/software/arlequin3).

## Additional Information

**How to cite this article:** Ayo, C. M. *et al*. Ocular toxoplasmosis: susceptibility in respect to the genes encoding the KIR receptors and their HLA class I ligands. *Sci. Rep*. **6**, 36632; doi: 10.1038/srep36632 (2016).

**Publisher’s note:** Springer Nature remains neutral with regard to jurisdictional claims in published maps and institutional affiliations.

## Figures and Tables

**Table 1:** General characteristics and serological profile of patients with and without ocular toxoplasmosis and its primary or recurrent manifestations.

Characteristic	Patients without ocular toxoplasmosis* (n = 149)	Patients with ocular toxoplasmosis** (n = 148)	Patients with primary manifestation (n = 120)	Patients with recurrent manifestation (n = 28)
Age (Mean ± SD)	57.7 ± 16.9ª,^b^,^c^	42.3 ± 20.6ª	44.9 ± 20.9^b^,^d^	31.8 ± 30.5^c^,^d^
Median	60	37	46	30
Gender N (%)				
Female	76 (51.0)	69 (46.6)	55 (45.8)	14 (50.0)
Male	73 (49.0)	79 (53.4)	65 (54.2)	14 (50.0)
Serological profile N (%)				
IgM−/IgG+	149 (100.0)	143 (96.6)	115 (95.8)	28 (100.0)
IgM +/IgG+	0 (0.0)	5 (3.4)	5 (4.2)	0 (0.0)
IgM +/IgG−	0 (0.0)	0 (0.0)	0 (0.0)	0 (0.0)

Clinical diagnosis: ^*^Presence of ocular diseases other than toxoplasmosis as cataracts (17.5%), pterygium (4.0%), agerelated macular degeneration (23.0%), glaucoma (6.7%), retinal detachment (16.1%), optic neuropathy (3.4%), macular edema (4.6%), macular atrophy (2.6%), diabetic retinopathy (8.7%) and other ocular diseases (13.4%). ^**^Presence of ocular scars/lesions due to toxoplasmosis. t = Student t test.

^a^*P < *0.0001 t = 7.00 (Patients without ocular toxoplasmosis vs. Patients with ocular toxoplasmosis).

^b^*P < *0.0001 t = 5.48 (Patients without ocular toxoplasmosis vs. Patients with primary manifestation).

^c^*P < *0.0001 t = 7.51 (Patients without ocular toxoplasmosis vs. Patients with recurrent manifestation).

^d^*P =* 0.002 t = 3.12 (Patients with primary manifestation vs. Patients with recurrent manifestation).

**Table 2:** Distribution of KIR genes and KIR genotypes in patients with and without ocular toxoplasmosis and its primary or recurrent manifestations.

Genes	Patients without ocular toxoplasmosis (n = 149)	Patients with ocular toxoplasmosis (n = 148)	Patients with primary manifestation (n = 120)	Patients with recurrent manifestation (n = 28)
	N (%)	N (%)	N (%)	N (%)
*KIR2DL1*	149 (100)	146 (98.6)	118 (98.3)	28 (100)
*KIR2DL2*	95 (63.8)	101 (68.2)	75 (62.5)	23 (82.1)
*KIR2DL3*	131 (87.9)	128 (86.5)	102 (85.0)	26 (92.9)
*KIR2DL4*	149 (100)	148 (100)	120 (100)	28 (100)
*KIR2DL5*	85 (57.0)	93 (62.8)	70 (58.3)	23 (82.1)
*KIR2DP1*	149 (100)	146 (98.6)	118 (98.3)	28 (100)
*KIR2DS1*	63 (42.3)	66 (44.6)	51 (42.5)	15 (53.6)
*KIR2DS2*	40 (26.8)^a^	25 (16.9)^a^	20 (16.6)	5 (17.9)
*KIR2DS3*	54 (36.2)	58 (39.2)	42 (35.0)	16 (57.1)
*KIR2DS4*	144 (96.6)	141 (95.2)	113 (94.2)	28 (100)
*KIR2DS5*	56 (37.6)	62 (41.9)	49 (40.8)	13 (46.4)
*KIR3DL1*	143 (96.0)	145 (98.0)	117 (97.5)	28 (100)
*KIR3DL2*	149 (100)	148 (100)	120 (100)	28 (100)
*KIR3DL3*	149 (100)	148 (100)	120 (100)	28 (100)
*KIR3DP1*	149 (100)	148 (100)	120 (100)	28 (100)
*KIR3DS1*	39 (26.1)^b^,^c^	64 (43.2)^b^	49 (40.8)	15 (53.6)^c^
**Genotypes**				
AA	41 (27.5)	33 (22.3)	27 (22.5)	6 (21.4)
BX	108 (72.4)	115 (77.7)	93 (80.0)	22 (78.5)

^a^OR = 0.55; CI = 0.31–0.97; *P* = 0.05; *Pc* = 0.80 (Patients without ocular toxoplasmosis vs. Patients with ocular toxoplasmosis).

^b^OR = 2.15; CI = 1.31–3.50; *P* = 0.003, *Pc* = 0.04 (Patients without ocular toxoplasmosis vs. Patients with ocular toxoplasmosis).

^c^OR = 3.25; CI = 1.42–7.44, *P* = 0.007; *Pc* = 0.1 (Patients without ocular toxoplasmosis vs. Patients with recurrent manifestation).

**Table 3:** Distribution of the HLA class I KIR-ligands in in patients with and without ocular toxoplasmosis and its primary or recurrent manifestations.

HLA ligands*	Patients without ocular toxoplasmosis (n = 149)	Patients with ocular toxoplasmosis (n = 148)	Patients with primary manifestation (n = 120)	Patients with recurrent manifestation (n = 28)
	N (%)	N (%)	N (%)	N (%)
A3 and/or A11	31 (20.8)	42 (28.4)	32 (26.7)	10 (35.7)
Bw4	113 (75.8)	104 (70.3)	84 (70.0)	20 (71.4)
Bw4-80Ile	93 (62.4)	87 (58.8)	72 (60.0)	15 (53.6)
Bw4-80Thr	46 (30.9)	39 (26.4)	29 (24.2)	10 (35.7)
C1C2	62 (41.6)	58 (39.2)	46 (38.3)	12 (42.9)
C1C1	26 (17.4)	25 (16.9)	19 (15.8)	6 (21.4)
C2C2	61 (40.9)	65 (43.9)	55 (45.8)	10 (35.7)

Bw4 = HLA-A*23, *24, *32; HLA-B *13, *27, *44, *51, *52, *53, *57, *58. Bw4-80Ile = HLA-A*23, *24, *32; HLA-B*51, *52, *53, *57, *58. Bw4-80Thr = HLA-B *13, *27, *44. Group C1 = HLA-C*01, *03, *07, *08, *12, *14, *16. Group C2 = HLA-C*02, *04, *05, *06, *07, *15, *17, *18. ^*^The same individual could express more than one pair KIR-HLA ligand.

**Table 4:** Distribution of KIR and their respective HLA ligands in patients with and without ocular toxoplasmosis and its primary or recurrent manifestations.

KIR - HLA ligands	Patients without ocular toxoplasmosis (n = 149)	Patients with ocular toxoplasmosis (n = 148)	Patients with primary manifestation (n = 120)	Patients with recurrent manifestation (n = 28)
	N (%)	N (%)	N (%)	N (%)
2DL1-C2	123 (82.6)	121 (81.8)	99 (82.5)	22 (78.6)
2DL2-C1	55 (36.9)	57 (38.5)	41 (34.2)	16 (57.1)
2DL3-C1	79 (53.0)	71 (48.0)	55 (45.8)	16 (57.1)
3DL2-A3/A11	31 (20.8)	42 (28.4)	32 (26.7)	10 (35.7)
3DL1-Bw4	109 (71.2)	101 (68.2)	82 (68.3)	19 (67.8)
3DL1-Bw4-80Ile	90 (60.4)	81 (54.7)	68 (56.6)	13 (46.4)
3DL1-Bw4-80Thr	45 (30.2)	38 (25.7)	28 (23.3)	10 (35.7)
2DS1-C2	49 (32.9)	55 (37.2)	44 (36.7)	11 (39.3)
2DS2-C1	22 (14.8)	16 (10.8)	11 (9.2)	5 (17.9)
3DS1-Bw4-80Ile	19 (12.8)^a^,^b^,^c^	37 (25.0)^a^	28 (23.3)^b^	9 (32.1)^c^
2DL1-C2C2	61 (40.9)	63 (42.6)	53 (44.2)	10 (35.7)
2DL2-C1C1	19 (12.8)	20 (13.5)	14 (21.4)	6 (21.4)
2DL3-C1C1	23 (15.4)	23 (15.5)	17 (14.2)	6 (21.4)
2DS1-C2C2	18 (12.1)	31 (20.9)	26 (21.7)	5 (17.9)
2DS2-C1C1	10 (6.7)	4 (2.7)	2 (1.7)	2 (7.1)
2DL2/2DL2-C1C2	6 (4.0)	10 (6.8)	8 (6.7)	2 (7.1)
2DL2/2DL3-C1C2	30 (20.1)	27 (18.2)	19 (15.8)	8 (28.6)
2DL3/2DL3-C1C2	56 (37.6)^d^,^e^,^f^	21 (14.2)^d^	19 (15.8)^e^	2 (7.1)^f^
2DL2/2DL2-C1C1	3 (2.0)	2 (1.4)	2 (1.7)	0 (0.0)
2DL2/2DL3-C1C1	16 (10.7)	18 (12.2)	12 (10.0)	6 (21.4)
2DL3/2DL3-C1C1	23 (15.4)^g^,^h^,^i^	5 (3.4)^g^	5 (4.2)^h^	0 (0.0)^i^

^a^OR = 2.28; CI = 1.24–4.19; *P* = 0.007; *Pc* = 0.01 (Patients without ocular toxoplasmosis vs. Patients with ocular toxoplasmosis).

^b^OR = 2.08; CI = 1.09–3.95; *P* = 0.02; *Pc* = 0.04 (Patients without ocular toxoplasmosis vs. Patients with primary manifestation).

^c^OR = 3.24; CI = 1.28–8.89; *P* = 0.02; *Pc* = 0.04 (Patients without ocular toxoplasmosis vs. Patients with recurrent manifestation).

^d^OR = 0.27; CI = 0.15–0.48; *P* = 0.000007; *Pc* = 0.00002 (Patients without ocular toxoplasmosis vs. Patients with ocular toxoplasmosis).

^e^OR = 0.31; CI = 0.17–0.56; *P* = 0.0001; *Pc* = 0.0003 (Patients without ocular toxoplasmosis vs. Patients with primary manifestation).

^f^OR = 0.12; CI = 0.29–0.59; *P* = 0.003; *Pc* = 0.009 (Patients without ocular toxoplasmosis vs. Patients with recurrent manifestation).

^g^OR = 0.19; CI = 0.07–0.51; *P* = 0.0005; *Pc* = 0.001 (Patients without ocular toxoplasmosis vs. Patients with ocular toxoplasmosis).

^h^OR = 0.23; CI = 0.08–0.64; *P* = 0.002; *Pc* = 0.006 (Patients without ocular toxoplasmosis vs. Patients with primary manifestation).

^i^OR = 0.09; CI = 0.05–0.91; *P* = 0.01; *Pc* = 0.05 (Patients without ocular toxoplasmosis vs. Patients with recurrent manifestation).

**Table 5:** Distribution of activating KIR plus inhibitory KIR and their respective ligands in patients with and without ocular toxoplasmosis and its primary or recurrent manifestations.

KIR - HLA ligands	Patients without toxoplasmic retinochoroiditis (n = 149)	Patients with toxoplasmic retinochoroiditis (n = 148)	Patients with primary manifestation (n = 120)	Patients with recurrent manifestation (n = 28)
	N (%)	N (%)	N (%)	N (%)
KIR-C1				
2DS2+/2DL2−/C1+	0 (0.0)	0 (0.0)	0 (0.0)	0 (0.0)
2DS2−/2DL2+/C1+	33 (22.1)	41 (27.7)	30 (25.0)	11 (39.3)
2DS2+/2DL3−/C1+	9 (6.0)	12 (8.1)	10 (8.3)	2 (7.1)
2DS2−/2DL3+/C1+	66 (44.3)	67 (45.3)	54 (54.0)	13 (46.4)
2DS2+/2DL2−/2DL3−/C1+	0 (0.0)	0 (0.0)	0 (0.0)	0 (0.0)
2DS2−/2DL2−/2DL3+/C1+	33 (22.1)	26 (17.6)	24 (20.0)	2 (7.1)
2DS2+/2DL2−/2DL3+/C1+	0 (0.0)	0 (0.0)	0 (0.0)	0 (0.0)
2DS2−/2DL2+/2DL3+/C1+	33 (22.1)	41 (27.1)	30 (25.0)	11 (39.3)
2DS2+/2DL2+/2DL3-/C1+	9 (6.0)	12 (8.1)	10 (8.3)	2 (7.1)
2DS2+/2DL2+/2DL3+/C1+	13 (8.7)	4 (2.7)	4 (3.3)	0 (0.0)
KIR-C2				
2DS1+/2DL1−/C2+	0 (0.0)	2 (1.4)	2 (1.7)	0 (0.0)
2DS1−/2DL1+/C2+	74 (49.7)	67 (45.3)	56 (46.7)	11 (39.3)
2DS1+/2DL1+/C2+	49 (32.9)	54 (36.5)	43 (35.8)	11 (39.3)
KIR-BW4-80Ile				
3DS1+/3DL1+/BW4−80Ile+	32 (21.5)	53 (35.8)	42 (35.0)	11 (39.3)
3DS1+/3DL1−/BW4−80Ile+	1 (0.7)	3 (2.0)	3 (2.5)	0 (0.0)
3DS1−/3DL1+/BW4−80Ile+	71 (47.7)^a^,^b^	48 (32.4)^a^	45 (37.5)^c^	3 (10.7)^b^,^c^

^a^OR = 0.52; CI = 0.32–0.84; *P* = 0.009; *Pc* = 0.02 (Patients without ocular toxoplasmosis vs. Patientes with ocular toxoplasmosis).

^b^OR = 0.13; CI = 0.03–0.45; *P* = 0.0003; *Pc* = 0.0009 (Patients without ocular toxoplasmosis vs. Patientes with recurrent manifestation).

^c^OR = 0.20; CI = 0.05–0.70; *P* = 0.006; *Pc* = 0.01 (Patients with primary manifestation vs. Patients with recurrent manifestation).
